# The effect of ear acupressure on occupational cognitive failure in nurses after the COVID-19 crisis: a randomized controlled clinical trial

**DOI:** 10.1186/s12912-024-02139-w

**Published:** 2024-07-05

**Authors:** Mahsa Ovliaei Bidgoli, Zahra Tagharrobi, Khadijeh Sharifi, Zahra Sooki, Mansooreh Momen-Heravi, Fatemeh Zare Joshaghani, Mohammad Zare

**Affiliations:** 1 Trauma Nursing Research Center, Kashan University of Medical Sciences, Kashan, Iran; 2 Infectious Diseases Research Center, Kashan University of Medical Sciences, Kashan, Iran

**Keywords:** Auriculotherapy, Ear acupressure, Nurse, Cognitive failure

## Abstract

**Background:**

In the aftermath of the COVID-19 pandemic, nurses reported varying degrees of cognitive failure. To prioritize patient safety in clinical settings, it is important and necessary to address and mitigate the symptoms of cognitive failure among nurses.

**Aim:**

This study was conducted in Iran to evaluate the impact of ear acupressure on occupational cognitive failure in nurses.

**Methods:**

This randomized controlled clinical trial was conducted with 54 nurses who experienced cognitive failure in 2022. Sampling was performed by convenience. Fifty-six nurses who scored 40 or higher on the occupational cognitive failure questionnaire were randomly assigned to either the intervention group (28 subjects) or the sham group (28 subjects). In the intervention group, pressure was applied to the shen-men point, zero point, hippocampus, master cerebral, brain, and memory 1 and 2 of the earlobes for six weeks using Vaccaria seeds. In the sham group, a sticker without seeds was applied at the same points as in the intervention group, and no pressure was applied. Cognitive failure was assessed at the beginning of the study (T0), at the end of the intervention (sixth week of study, T1), and four weeks after the end of the intervention (tenth week of study, T2). The data were collected using contextual data questionnaires and the Occupational Cognitive Failure Questionnaire (OCFQ). The data obtained from 54 nurses (28 in the sham group and 26 in the intervention group) were analyzed by SPSS v16 using repeated-measures ANOVA.

**Results:**

The two groups had no significant differences regarding background variables. The between-group analysis revealed a significant interaction effect of time and intervention on cognitive failure (F = 60.320, *p* < 0.001, effect size = 0.537). The cognitive failure score in the intervention group was significantly lower at the end of the intervention and one month later than that in the sham group (*p* < 0.001). Within-group analysis revealed a significant difference in the cognitive failure scores of the intervention group at T0, T1, and T2 (61.231 ± 14.230, 34.000 ± 14.659, and 29.808 ± 14.266, respectively; F = 52.331, *p* < 0.001, effect size = 0.677). However, in the sham group, the cognitive failure score exhibited a brief but significant increase at T0, T1, and T2 (54.786 ± 11.239, 56.250 ± 10.950, and 56.000 ± 11.337, respectively; F = 6.369, *p* = 0.006, effect size = 0.191).

**Conclusion:**

Auriculotherapy has shown promise in improving occupational cognitive failure in nurses. It is recommended that nurses consider incorporating auriculotherapy as a complementary treatment modality, particularly through self-treatment programs, when experiencing symptoms of cognitive impairment.

**Trial Registration Number (TRN):**

IRCT20100211003329N10

**Date of registration:**

04/11/2022

**Supplementary Information:**

The online version contains supplementary material available at 10.1186/s12912-024-02139-w.

## Background

Cognitive function encompasses mental processes such as attention, perception, thinking, learning, and memory. Any disruption in these processes is referred to as cognitive failure [1]. Cognitive failure refers to errors in perception, memory, or attention that hinder an individual’s ability to perform tasks they would normally be capable of [2]. Nurses, particularly those working in intensive care units, are more susceptible to experiencing reduced attention, concentration, and cognitive changes due to high levels of stress, anxiety, depression, and heavy workloads compared to those in many other professions [3]. During the COVID-19 pandemic, nurses may face additional challenges, including contracting the disease, witnessing frequent deaths, fear of contagion, concerns about spreading the disease to their families, continuous work pressure, physical fatigue, and prolonged emotional strain. These factors contribute to increased stress, anxiety, and depression, ultimately leading to a significant percentage of nurses experiencing difficulties with concentration, attention, memory, information processing, and overall performance [4]. Supporting this notion, a study by Arnetz et al. (2021) conducted in the U.S. demonstrated that approximately 77% of nurses experienced varying degrees of cognitive impairment, with a more pronounced impact observed in specialized care and emergency departments [5]. Omar et al.‘s study in Egypt showed a strong negative effect of COVID-19 on memory and attention in healthcare workers who recovered from COVID-19 [6]. In Iran, a decrease in concentration has been reported as one of the most significant psychological consequences of COVID-19 among nurses [7].

Cognitive failure in nurses significantly affects individuals and their performance, leading to disruptions in daily activities. It results in weakened social interactions, reduced psychomotor performance, and decreased quality of life [1]. Moreover, cognitive failure has a negative impact on the nursing care provided. It can lead to unfortunate incidents and increase the risk of clinical errors, jeopardizing patient safety and potentially causing financial and even life-threatening consequences [5].

Currently, the primary approach to improving cognitive function involves the use of stimulants. However, the side effects associated with attention-stimulating drugs, such as decreased appetite, sleep disorders, headaches, abdominal discomfort, increased lethargy, and fatigue, make them less suitable for widespread use [8, 9]. As a result, researchers have focused on exploring complementary therapies in recent years to address cognitive function issues. These therapies include cognitive-behavioral interventions [10], acupressure [11], acupuncture [12], herbal medicines [13], mechanical massage [14], and aromatherapy [15]. However, it is worth noting that most of these studies have primarily targeted the elderly population with cognitive failure, and none of the studies have specifically examined cognitive failure related to specific job categories or work environments. Furthermore, considering the working conditions of nurses, factors such as time constraints, limited accessibility, and the need to perform some of these interventions in specific settings pose challenges in implementing these interventions within this particular group.

One of the nonpharmacological methods that has been introduced and is available, affordable, without significant complications, and suitable for different time and place conditions is ear acupressure or auriculotherapy [16]. Several studies have investigated the effects of this method on cognitive function in individuals with dementia [17] and on attention and concentration in children with attention deficits [17, 18]. Ear acupressure is a noninvasive and well-accepted technique for patients. In this method, the external part of the human ear is viewed as an inverted embryo, with the head of the fetus represented by the earlobe and the hands and feet positioned upward. The remaining organs of the fetus are represented by different points on the external ear. A standardized naming system was used to identify points on various regions of the right and left external ear anatomy [16]. When there is a disturbance or dysfunction in a specific area of the body, the corresponding area on the outer ear becomes more sensitive. In some cases, there may even be changes in the color and morphology of the corresponding area. It is believed that by stimulating specific points on the external ear, the organ associated with that stimulated area can be influenced [19]. For example, Olson suggested that the Shen Men, Point Zero, Master Cerebral Point, Memory 1 and 2, Brain, and Hippocampus points are related to cognitive status, and their stimulation can improve memory, attention, and concentration problems [19]. The exact mechanism of action for ear acupressure is not fully understood. Ear acupressure may activate energy channels, regulate and balance energy flow in the body, and impact hormone levels and the release of neurotransmitters such as serotonin. It also improves blood circulation, induces deep relaxation, and stimulates and modulates brain activity [20]. Furthermore, due to the effect on neurotransmitters and improving blood circulation, this hypothesis has proposed that ear acupressure may promote the growth and development of brain nerve fibers, increase the number of nerve synapses, and enhance their function within the brain cortex, thereby affecting concentration and attention [19].

Based on the data available in the databases, a specific study focusing on the effect of ear acupressure on cognitive failure or related variables in occupational groups such as nurses or individuals who have recovered from the acute stage of COVID-19 has not been conducted within or outside Iran. However, a limited number of other existing studies that are most relevant to this topic have explored the impact of ear acupressure on children with attention deficit hyperactivity disorder (ADHD) or cognitive function in individuals with dementia [16, 18]. Two studies conducted in Iran have shown positive effects of ear acupressure on improving attention in children with attention deficit disorder [16, 18]. However, it is important to note that the outcome variables and target groups investigated in these studies differ from those considered in the present study. A systematic review conducted by Kwon et al. (2018) included nine articles on the effect of ear acupressure on cognitive function in patients with dementia. Partially effective interventions for cognitive function were reported in patients with mild and moderate dementia. However, the review indicated a lack of significant effects in patients with vascular dementia. It is worth mentioning that the authors of this review acknowledged the limitations of the included studies, such as the small number of articles and weak methodology. Thus, they emphasized the need for further investigations in this area [17].

Therefore, considering the importance of cognitive impairment as one of the leading long-term consequences of the COVID-19 epidemic crisis for nurses [21], the lack of a similar study, the possible influence of the effect of auricular pressure medicine on the cause and nature of cognitive impairment [17], and the impossibility of extracting valid and reliable findings from previous related studies [18] are necessary to study the effect of ear acupressure on occupational cognitive impairment in nurses.

### Study Aim

The present study focused on evaluating the effect of ear acupressure on occupational cognitive failure among Iranian nurses after the COVID-19 crisis.

## Methods

### Study type and design

The present study was a randomized controlled clinical trial conducted in 2022. The study population comprised nurses with a university education who were employed in clinical activities at Shahid Beheshti Hospital, Kashan’s largest educational and treatment center.

### Randomization, masking, and participant selection

The inclusion criteria for the study were as follows: employment in an inpatient department involving direct patient care, a minimum of 2 years of work experience in the hospital, willingness to participate in the study, possession of a university degree in nursing, absence of known chronic physical diseases or psychological disorders as self-reported by the individual, moderate to high cognitive failure (scoring 40 or higher on the Occupational Cognitive Failure Questionnaire), no skin sensitivity to alcohol and glue, no use of acupressure or acupuncture in the last three months, good health in the earlobe area, not currently being pregnant, no use of cigarettes or drugs, or alcohol as self-reported by the individual, and not undergoing medical treatment aimed at improving cognitive status. The exclusion criteria included inability to access the participant during the study (e.g., due to emigration or death), hospitalization during the study period, withdrawal of cooperation during the study, experience of acute and severe stress events (e.g., death of loved ones, divorce) during the study, allergy or intolerance to glue (evidenced by allergy symptoms or redness), presence of a severe and acute physical or mental illness during the study, failure to comply with the criteria regarding the daily average duration of pressure on all points in each week of the intervention, and concurrent use of other nonpharmacological methods and treatments.

The researchers used convenience sampling with random allocation to groups in their study. The article’s first author went to Shahid Beheshti Hospital in Kashan and identified the nurses who met the inclusion criteria. To determine eligibility, each nurse completed the occupational cognitive failure questionnaire. Those who scored 40 or higher on the questionnaire were considered to have moderate to high cognitive impairment. After providing consent, the eligible participants were listed in the order of examination. The allocation of participants to the intervention group and control group (sham) was performed using block randomization, which involved the use of blocks of 4 and 8. The researchers utilized the online software provided by Sealed Envelope Ltd. (2017), available through www.sealedenvelope.com. A total of 9 blocks were defined, consisting of 4 blocks of 4 participants and five blocks of 8 participants. The allocation of participants to the groups was determined based on the order specified by the software.

### Sample size calculation

The sample size was determined on the basis of Formula 1. Based on the Raeisi et al. (2018) study the standard deviation was set at 0.61 [9]. In addition, the parameters effect size 0.5 [22], test power of 80%, and confidence level of 95% were considered. Using Formula 1, the sample size was calculated to be 24 subjects for each group. However, considering a probable dropout rate of approximately 15%, the sample size was adjusted accordingly, and 28 participants were included in each group.

Formula 1 $$n=\frac{2{\left({Z}_{1-\raisebox{1ex}{$\alpha $}\!\left/ \!\raisebox{-1ex}{$2$}\right.}+{Z}_{1-\beta }\right)}^{2}{\delta }^{2}}{{d}^{2}}$$

### Ear acupressure

**Fig. 1:**
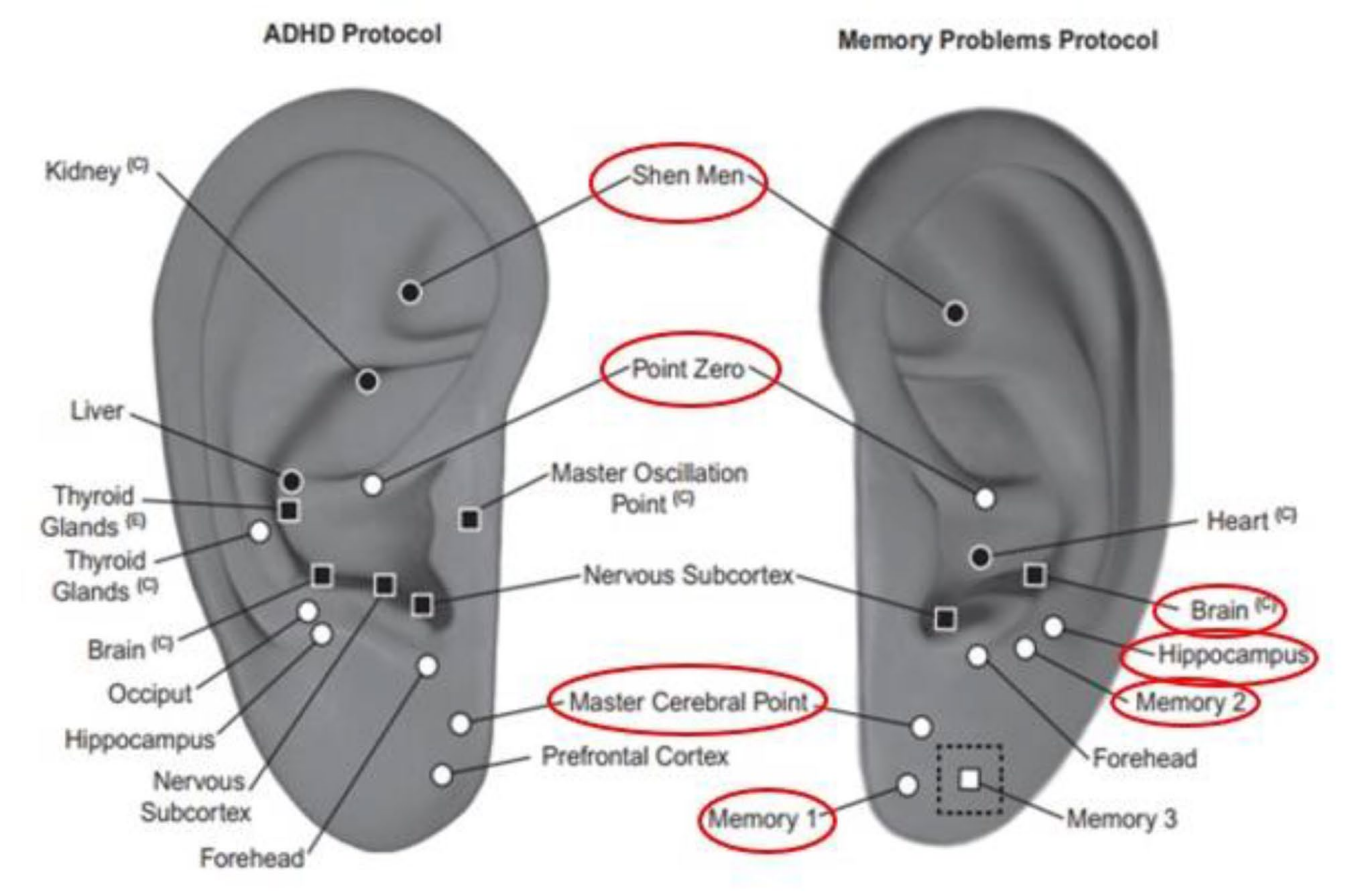
Points related to cognitive function

The article’s first author, a nursing master’s student, received training under the supervision of a specialist in ear acupressure (F Z-J, sixth author), who served as a project partner. After learning the theoretical and practical aspects of the method and spotting in 10 sessions, she identified the relevant points in 15 clients using the map of the points on the ear, and the correctness of the spotting she performed was confirmed by the specialist (F Z-J) in the last 10 clients; Therefore, her ability was confirmed to perform the intervention. In the intervention group, the researcher applied Vaccaria seeds (ZHONG YANG mark made in China) to 7 specific points associated with cognitive function [23]. These points included the Shen Men, zero point, hippocampus, master cerebral, brain, and memory 1 and 2 points (Fig. 1) [19]. The nurses were taught to stimulate these points using their thumb and forefinger for six weeks, five days a week, and three times a day, and each point was pressed for one minute [17]. In the sham group, adhesives were applied to the designated points without seeds, and no pressure was applied at these points. It is important to note that throughout the 6-week intervention period, the researcher applied Vaccaria seeds to the intervention group and adhesives without seeds to the sham group, alternating between the left and right ears each week. The alternate use of both ears was done based on the recommendation of some Iranian traditional medicine experts, who believe that the use of the right or left ear is effective depending on a person’s temperament. Before application, the researcher cleaned the skin with an alcohol cotton swab. The seeds were placed on the ears five days a week, and the participants were instructed to remove the adhesives at the end of the fifth day to prevent potential allergic reactions. If the adhesives detached during the five days, the participants were advised to contact the researcher, who would then reapply the seeds to the ears in an agreed-upon location.

During the intervention, a weekly note sheet was provided to the participants. This sheet recorded the number of times and duration of pressure applied to each point during each session and any occurrences of allergic reactions or the initiation of new medication. Every week during the intervention period, all participants were visited at a predetermined location, new seeds were applied, their notes were collected, and new sheets were given to them for the following week. If the average duration of pressure at all points in the intervention group during each week of the intervention was less than 14 min per day, that individual was excluded from the study. The application of seeds continued for six weeks, and the participants were followed up for four weeks after the intervention ended. To assess the intervention’s effect and long-term effects after ceasing it, the participants completed the Occupational Cognitive Failure Questionnaire three times: at the beginning of the study (T0), immediately after the end of the intervention (sixth week of study; T1), and four weeks after the end of the intervention (tenth week of study; T2). The participants’ workplace addresses and phone numbers were recorded during sampling. Throughout the intervention period, at the end of each week, as well as during the 4-week follow-up, phone calls were made to confirm the time and location of the visit. If necessary, transportation arrangements were made with the participants. The entire sampling and data collection process took place from November 22, 2022, to February 20, 2023 (3 months).

### Instruments

1) The background data questionnaire: This tool consists of various variables, including age, sex, education, marital status, number of children, place of residence, belonging to Kashan city by birth or origin, housing status, nursing work experience, ward of service, amount of monthly overtime, type of employment, work shift, occupational position, engagement in nursing activity as a second job, engagement in nonnursing activity as a second job, interest in the nursing profession, history of using any type of complementary therapy, and history of using auricular acupressure. The qualitative content validity of this questionnaire was confirmed by six faculty members from the Kashan School of Nursing and Midwifery.

2) Occupational Cognitive Failure Questionnaire (OCFQ): This questionnaire was developed by Hassanzadeh Rangi et al. in 2012. It consists of 30 items that are rated on a five-point Likert scale ranging from 0 to 4 (0 = never, 1 = rarely, 2 = moderate, 3 = a lot, and 4 = very much). The items assess memory, attention, and performance-related issues in individuals, such as forgetting phone numbers or names and making mistakes in job duties. The questionnaire is divided into three dimensions: memory (questions 1–10), attention (questions 11–20), and performance (questions 21–30). The total score on the questionnaire ranges from 0 to 120, with higher scores indicating a greater degree of cognitive failure. Hassanzadeh Rangi et al. confirmed the scale’s content validity. Its Cronbach’s alpha coefficient was also reported to be 0.96 [24]. In the present study, the questionnaire’s internal consistency was estimated to be 0.884 using Cronbach’s alpha.

3) Daily note sheet: This sheet was designed explicitly for weekly recording purposes. It captured information regarding the frequency and duration of pressure applied to the points, any instances of local sensitivity, and the nurse’s medication use.

### Statistical analysis

The data analysis was conducted using SPSS version 16 statistical software (SPSS Inc, Chicago, IL, USA). If necessary, categorical variables were compared between the two groups using the chi-square or Fisher’s tests. For quantitative variables between two groups, the independent t-test was utilized, and if the data did not follow a normal distribution, the nonparametric equivalent Mann‒Whitney U test was used. Repeated measures ANOVA was used to compare cognitive failure scores within and between groups. Despite the small number of samples dropped, an intention-to-treat (ITT) analysis was also performed to improve the quality of the studies, in addition to a per-protocol (PP) analysis. The normality of the quantitative data was assessed using skewness and kurtosis indices, considering a range of ± 2 as indicative of a normal distribution. A significance level of less than 0.05 was used to indicate statistical significance in all the patients.

## Results

**Fig. 2:**
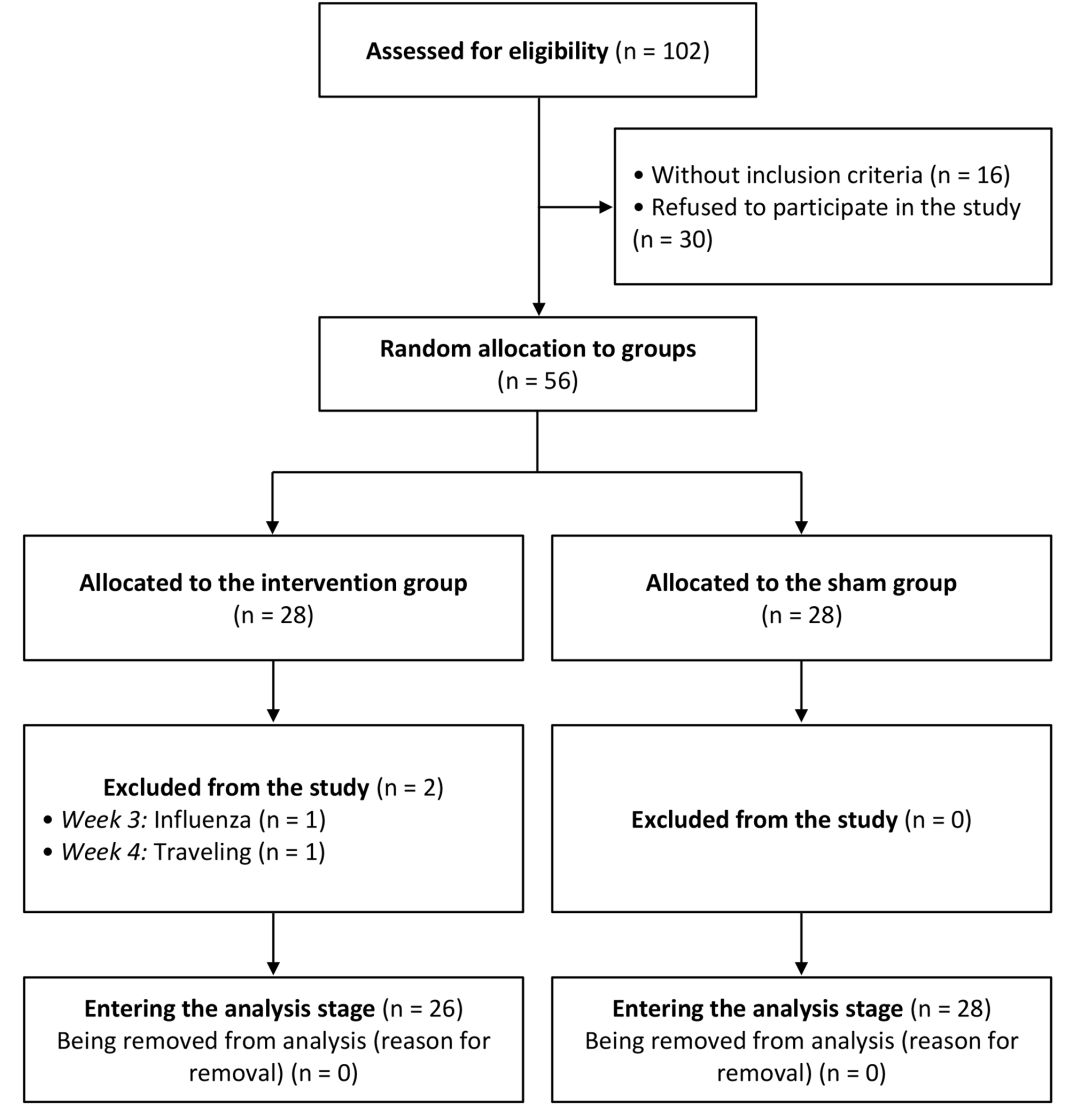
The flow diagram of the study

In the present study, 102 nurses were initially examined; 56 eligible participants were selected and assigned to two groups: the intervention group and the sham group (Fig. 2). There were no statistically significant differences between the intervention and sham groups regarding the background variables investigated (Table 1). Four participants (7.41%) reported experiencing mild local itching. None of the nurses exhibited severe local allergic symptoms during the study period. Furthermore, no significant difference was observed between the two groups regarding minor itching (*P* = 0.612).

**Table 1:** Between-group comparisons of participants’ baseline characteristics, Kashan, 2022

Group Variable		Intervention (*n* = 26)	Sham (*n* = 28)	*p* value
		Mean ± SD No (%)	Mean ± SD No (%)	
Age (Years)		31.500 ± 5.791	30.607 ± 5.606	0.567 ^a^
Sex	Female	24 (92.3)	20 (71.4)	0.079 ^b^
	Male	2 (7.7)	8 (28.6)	
Education	Bachelor’s Degree	25 (96.2)	25 (89.3)	0.612 ^b^
	Master’s Degree	1 (3.8)	3 (10.7)	
Marital Status	Single	8 (30.8)	7 (25)	0.636 ^d^
	Married	18 (69.2)	21 (75)	
Number of Children	0	15 (57.7)	17 (60.7)	0.928 ^c^
	1	7 (26.9)	8 (28.6)	
	2 or 3	4 (15.4)	3 (10.7)	
Belonging to Kashan City	Yes	25 (96.2)	24 (85.7)	0.353 ^b^
	No	1 (3.8)	4 (14.3)	
Place of Residence	Urban	24 (92.3)	28 (100)	0.227 ^b^
	Rural	2 (7.7)	0	
Housing Status	Owner	21 (80.8)	22 (78.6)	0.564 ^c^
	Rental	5 (19.2)	4 (14.3)	
	Relative’s Houses	0	2 (7.1)	
Job Position	Clinical Nurse	24 (92.3)	26 (92.9)	1 ^b^
	Supervisor	2 (7.7)	2 (7.1)	
Employment Status	Examinatory	1 (3.8)	0	0.435 ^c^
	Internship	5 (19.2)	9 (32.1)	
	Contractual	5 (19.2)	7 (25)	
	Regular Employment	15 (57.7)	12 (42.9)	
Ward	ICU	0	6 (21.4)	0.083 ^c^
	Emergency	20 (76.9)	15 (53.6)	
	Pediatric	2 (7.7)	1 (3.6)	
	Medical	3 (11.5)	3 (10.7)	
	Surgical	1 (3.8)	3 (10.7)	
Working Shift	Fixed	0	2 (7.1)	0.491 ^b^
	Rotating	26 (100)	26 (92.9)	
Overtime shift per month (hours)		66.192 ± 36.438	84.286 ± 44.426	0.063 ^e^
Work Experience (Years)		7.846 ± 5.425	7.214 ± 4.849	0.653 ^a^
Engaging in non-Nursing Activities as a Second Job	Yes	1 (3.8)	3 (10.7)	0.612 ^b^
	No	25 (96.2)	25 (89.3)	
History of Using Complementary Therapies	Yes	6 (23.1)	4 (14.3)	0.494 ^b^
	No	20 (76.9)	24 (85.7)	
History of Using Auriculotherapy	Yes	2 (7.7)	0	0.227 ^b^
	No	24 (92.3)	28 (100)	
Interest in Nursing (On A Scale Of 0 To 5)		3.500 ± 1.068	3.500 ± 1.621	1 ^a^

^a^ Independent t-Test

^b^ Fisher’s Exact Test

^c^ Exact type/Chi-Square

^d^ Chi-Square

^e^ Mann‒Whitney U Test

**Fig. 3:**
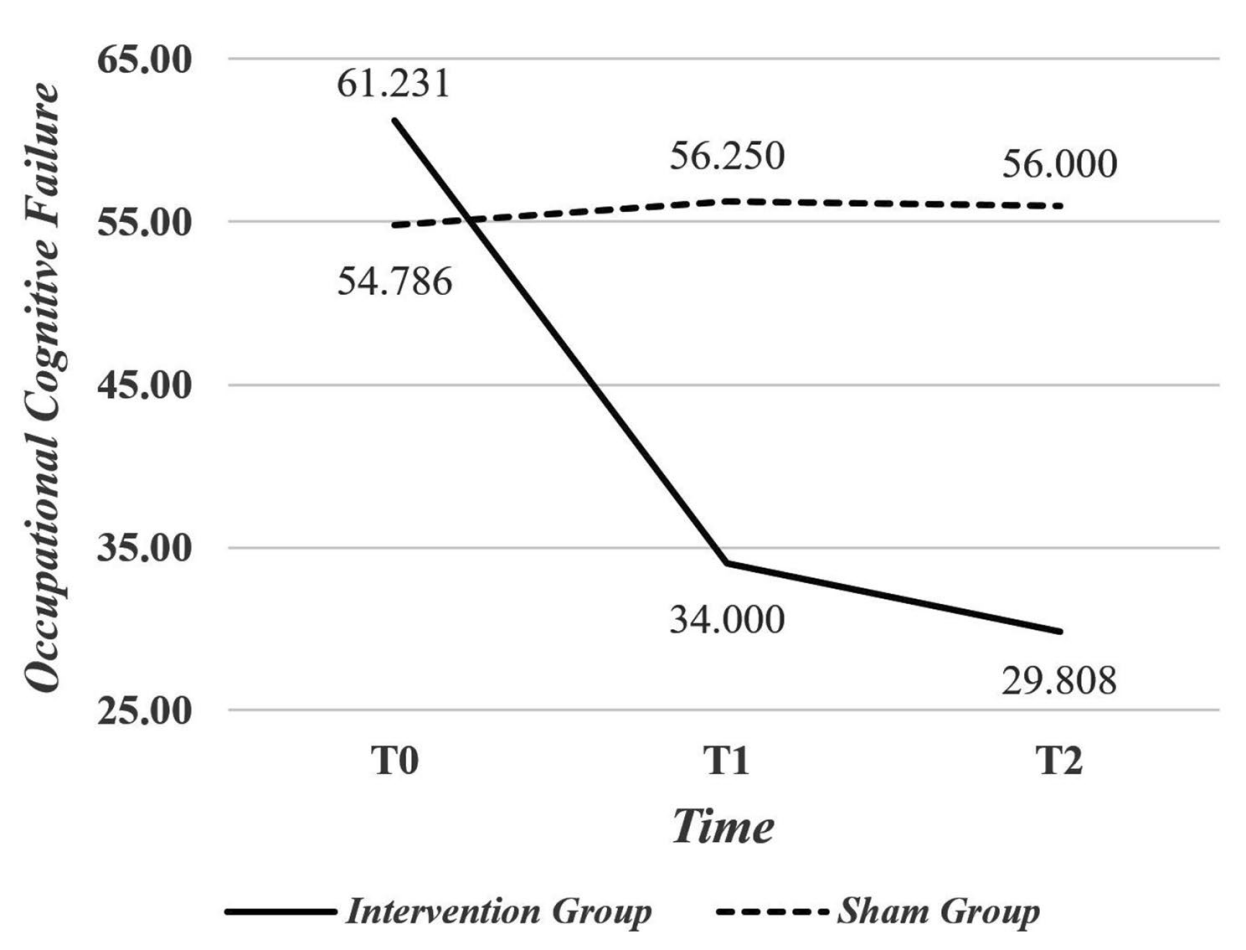
The cognitive failure scores in the two groups at the beginning (T0), end of the intervention (T1), and four weeks after the intervention (T2)

The interaction effect of time and intervention on the cognitive failure score was significant (F = 60.320, effect size = 0.537, *p* < 0.001) (Fig. 3; Table 2). In the intervention group, the effect of time on the cognitive failure score was statistically significant (F = 52.331, effect size = 0.677, *p* < 0.001). These findings indicate significant differences in the cognitive failure scores of the intervention groups at three different time points. In the sham group, the effect of time on the cognitive failure score was significant (F = 6.369, effect size = 0.191, *p* = 0.006) (Table 2). At each time point, the significance of the difference in the cognitive failure score between the intervention and sham groups was assessed using independent t-tests. The difference between the two groups was insignificant at T0 (*p* = 0.069). However, at the other time points, there was a significant difference between the two groups in terms of the cognitive failure score (*p* < 0.001) (Table 2).

**Table 2:** Within- and between-group comparisons of the mean score of occupational cognitive failure

Group OCF Score (0-120)			Sham (*n* = 28) Mean ± SD	Intervention (*n* = 26) Mean ± SD	Time-Group Interaction ^a^		Between Group Comparison ^b^
					Mauchly’s Test	Greenhouse‒Geisser	
Before the intervention (T0)			54.786 ± 11.239	61.231 ± 14.230	χ^2^ = 32.750 *p* < 0.001	F = 60.320 ES = 0.537 *p* < 0.001	*p* = 0.069
Six weeks after intervention onset (T1)			56.250 ± 10.950	34.00 ± 14.659			*p* < 0.001
ten weeks after intervention onset (T2)			56.000 ± 11.337	29.808 ± 14.266			*p* < 0.001
Within Group Comparison	Effect of Time		F = 6.369 ES = 0.191 *p* = 0.006	F = 52.331 ES = 0.677 *p* < 0.001	-		
	Pairwise Comparison ^c^	Difference T0 & T1	*p* < 0.001	*p* < 0.001			
		Difference T0 & T2	*p* = 0.040	*p* < 0.001			
		Difference T1 & T2	*p* = 1	*p* = 0.085			

ES: Effect Size

^a^ Analysis of Variance (ANOVA) with Repeated Measures

^b^ Independent t-test

^c^ Bonferoni Statistics

The interaction effect of time and intervention on the score of the memory dimension of cognitive failure was significant (F = 52.899, effect size = 0.504, *p* < 0.001). In the intervention group, there were statistically significant differences in the scores on the memory dimension at three different time points (F = 51.020, effect size = 0.671, *p* < 0.001). However, in the sham group, the effect of time on the memory dimension score was not significant (F = 0.618, *p* = 0.477). There was no significant difference in the scores on the memory dimension between the two groups at T0 (*p* = 0.283). However, significant differences were observed between the two groups at other time points (*p* < 0.001) (Table 3).

**Table 3:** Within- and between-group comparisons of the mean score on the memory dimension

Group Memory Dimension Score (0–40)			Sham (*n* = 28) Mean ± SD	Intervention (*n* = 26) Mean ± SD	Time-Group Interaction ^a^		Between Group Comparison ^b^
					Mauchly’s Test	Greenhouse‒Geisser	
Before the intervention (T0)			19.643 ± 4.637	21.154 ± 5.591	χ^2^ = 6.407 *p* = 0.041	F = 52.899 ES = 0.504 *p* < 0.001	*p* = 0.283
Six weeks after intervention onset (T1)			19.893 ± 4.740	12.654 ± 5.831			*p* < 0.001
ten weeks after intervention onset (T2)			20.00 ± 4.243	11.192 ± 5.404			*p* < 0.001
Within Group Comparison	Effect of Time		F = 0.618 *p* = 0.477	F = 51.020 ES = 0.671 *p* < 0.001	-		
	Pairwise Comparison ^c^	Difference T0 & T1	-	*p* < 0.001			
		Difference T0 & T2		*p* < 0.001			
		Difference T1 & T2		*p* = 0.256			

ES: Effect Size

^a^ Analysis of Variance (ANOVA) with Repeated Measures

^b^ Independent t-test

^c^ Bonferoni Statistics

The interaction effect of time and intervention on the score of the attention dimension of cognitive failure was found to be significant (F = 42.91, effect size = 0.452, *p* < 0.001). In the intervention group, the effect of time on the attention dimension score was significant (F = 39.828, effect size = 0.614, *p* < 0.001). In the sham group, the effect of time on the attention dimension score was not significant (F = 0.755, *p* = 0.183). Furthermore, there was no significant difference in the attention dimension score between the two groups at T0 (*p* = 0.094). However, significant differences were observed between the two groups at other time points (*p* < 0.001) (Table 4).

**Table 4:** Within- and between-group comparisons of the mean scores on the attention dimension

Group Attention Dimension Score (0–40)			Sham (*n* = 28) Mean ± SD	Intervention (*n* = 26) Mean ± SD	Time-Group Interaction ^a^		Between Group Comparison ^b^
					Mauchly’s Test	Greenhouse‒Geisser	
Before the intervention (T0)			18.857 ± 5.183	21.692 ± 6.973	χ^2^ = 23.907 *p* < 0.001	F = 42.910 ES = 0.452 *p* < 0.001	*p* = 0.094
Six weeks after intervention onset (T1)			19.321 ± 5.019	11.769 ± 5.771			*p* < 0.001
ten weeks after intervention onset (T2)			18.929 ± 5.263	9.615 ± 5.307			*p* < 0.001
Within Group Comparison	Effect of Time		F = 1.755 *p* = 0.183	F = 39.828 ES = 0.614 *p* < 0.001	-		
	Pairwise Comparison ^c^	Difference T0 & T1	-	*p* < 0.001			
		Difference T0 & T2		*p* < 0.001			
		Difference T1 & T2		*p* = 0.059			

ES: Effect Size

^a^ Analysis of Variance (ANOVA) with Repeated Measures

^b^ Independent t-test

^c^ Bonferoni Statistics

The interaction effect of time and intervention on the score of the performance dimension of cognitive failure was significant (F = 34.694, effect size = 0.400, *p* < 0.001). In the intervention group, the effect of time on the score on the functional dimension was significant (F = 28.328, effect size = 0.531, *p* < 0.001). In the sham group, the effect of time on the performance score was significant (F = 6.618, effect size = 0.197, *p* = 0.005). There was no significant difference in the performance dimension score between the two groups at T0 (*p* = 0.146). However, at other time points, the difference between the two groups was significant (*p* < 0.001) (Table 5).

**Table 5:** Within- and between-group comparisons of the mean score on the performance dimension

Group Performance Dimension Score (0–40)			Sham (*n* = 28) Mean ± SD	Intervention (*n* = 26) Mean ± SD	Time-Group Interaction ^a^		Between Group Comparison ^b^
					Mauchly’s Test	Greenhouse‒Geisser	
Before the intervention (T0)			16.286 ± 3.789	18.385 ± 6.413	χ^2^ = 49.625 *p* < 0.001	F = 34.694 ES = 0.400 *p* < 0.001	*p* = 0.146
Six weeks after intervention onset (T1)			17.036 ± 3.677	9.577 ± 4.500			*p* < 0.001
ten weeks after intervention onset (T2)			17.071 ± 3.800	9.000 ± 4.363			*p* < 0.001
Within Group Comparison	Effect of Time		F = 6.618 ES = 0.197 *p* = 0.005	F = 28.328 ES = 0.531 *p* < 0.001	-		
	Pairwise Comparison ^c^	Difference T0 & T1	*p* = 0.003	*p* < 0.001			
		Difference T0 & T2	*p* = 0.004	*p* < 0.001			
		Difference T1 & T2	*p* = 1	*p* = 1			

ES: Effect Size

^a^ Analysis of Variance (ANOVA) with Repeated Measures

^b^ Independent t-test

^c^ Bonferoni Statistics

The ITT analysis results were similar to those of the PP analysis. The ITT analysis revealed a significant interaction effect of time and intervention on the occupational cognitive impairment score (F = 49.238, effect size = 0.477, *p* < 0.001). In the intervention group, the effect of time was also significant (F = 45.655, effect size = 0.628, *p* < 0.001). A significant difference in occupational cognitive impairment score was observed between T0 and T1, as well as between T1 and T2 (*p* < 0.001). However, this score was not significantly different between T0 and T2 (*p* = 0.086).

In the sham group, the effect of time was significant (F = 6.369, effect size = 0.191, *p* = 0.006). There was a significant difference in the occupational cognitive impairment score between T0 and T1, as well as between T1 and T2, in this group (*p* < 0.001). However, this score was not significantly different between T0 and T2 (*p* = 1). There was no significant difference in occupational cognitive impairment scores between the two groups before the intervention (*p* = 0.069). However, this score was significantly greater in the sham group at T1 and T2 (*p* < 0.001).

## Discussion

The present study demonstrated the significant effect of auriculotherapy on cognitive failure in nurses. To the best of our knowledge, no previous study has specifically investigated the effect of auriculotherapy on occupational cognitive failure in any occupational category, including nursing. Therefore, the present study’s findings were compared to those of studies examining the effect of this intervention on attention, concentration, or cognition in different groups. Binesh et al.‘s study (2020) revealed a positive effect of auriculotherapy on attention levels in children with attention deficit hyperactivity disorder [18]. Additionally, a review conducted by Gao et al. (2023) supported the positive effect of auriculotherapy on cognitive failure and its dimensions in post-stroke patients [25]. Furthermore, a study conducted in Iran indicated that auriculotherapy improved the cognitive status of children with attention deficits [16]. Additionally, studies have confirmed the effectiveness of auriculotherapy on cognitive function in individuals with dementia [26, 27]. Considering the similarity of the variables investigated in these studies to the category of cognitive failure, despite differences in the target groups and the instruments used to measure cognitive status, it can be concluded that the findings of these studies align with the current study and support the positive effect of ear acupressure on cognitive status. In addition to revealing the positive effect of auriculotherapy, the present research also confirmed the effect of this intervention, which lasted at least one month. The findings of studies conducted on children with attention deficit disorder [16] and those conducted on stroke patients [12] further confirmed the long-lasting effect of this method, which is consistent with the findings of our study.

Auriculotherapy has been proposed to affect the brain and nervous system, possibly explaining its effectiveness on cognitive status and dimensions. It has been suggested that auriculotherapy can alter brain activity, leading to improvements in memory and attention while reducing activity in the dorsomedial prefrontal cortex [28, 29]. Furthermore, experts have observed that auriculotherapy can promote the growth and development of nerve-cerebral fibers and enhance the quantity and quality of nerve synapses in the cortex, potentially influencing cognitive function [14]. Studies focusing on the effect of auriculotherapy on cognitive function in individuals with dementia have suggested that this intervention leads to the release of endorphins and serotonin in the brain [26, 27]. It also promotes the production of internal compounds such as dopamine, noradrenaline, cortisol, and neuropeptides, which play a role in cognitive functioning [30]. It is generally believed that auriculotherapy stimulates the nervous system, triggering the release of neurotransmitters and inducing biochemical changes that can impact the overall homeostatic state of the body, leading to systemic improvements [31]. Considering these mechanisms, the positive role of auriculotherapy in managing cognitive failure in nurses can be justified. In other words, the underlying mechanisms proposed for auriculotherapy, particularly the integrated view of energetic and physiological mechanisms, can be used to explain the current research findings [30, 31].

In contrast to our study, a review showed a significant but weak effect of auriculotherapy on cognitive status in individuals with mild and moderate dementia, while no significant effect was observed in those with vascular dementia [17]. When considering the reasons for the discrepancy between the findings of Kwon et al.‘s study and the present study, as well as other similar studies, regardless of the difference in the characteristics of the target group, the instrument for assessing cognitive status and the type of treatment protocol used for auriculotherapy (points, how to apply pressure, the number of times and duration of pressure), methodological discussions should also be taken into account. The reason is that the authors of the mentioned review article admitted that their study could not be concluded due to the small number of articles, their weak methodology, and their inability to generalize the findings.

Notably, in the present study, the effect of time on the cognitive failure/performance dimension was significant in the sham group. However, this effect was not significant for the dimensions of memory and attention. In other words, in this group, there was a brief but significant increase in the total score of cognitive failure and the performance dimension over time. It is worth noting that the numerical values for the three dimensions of cognitive failure indicated that, at T0, the performance status was better than the memory and attention dimensions. This can be attributed to the fact that when facing difficult conditions and experiencing various physical and mental-psychological stresses, attention, concentration, and memory are initially disrupted, followed by a subsequent impact on performance [32]. This hypothesis can explain the slight increase in the score on the cognitive failure/performance dimension observed in the nurses in the sham group. However, it is important to consider certain factors that may have influenced the results. Thus, the continuation of the COVID-19 crisis throughout the study, the majority of the participants being employed in critical care and emergency wards, and challenging working conditions, including overcrowding and high workloads, should be considered.

Notably, experts have recognized that acupressure interventions have two components for therapeutic effects: random and special elements. During the study, the sham group did not experience the benefits of the special elements related to the performance of pressure on acupressure points. However, it is impossible to eliminate the random elements of this method in this group, which includes forming a therapeutic relationship between the patient and the therapist (such as empathy, attention, talking, and listening) during the sessions of sticking adhesives without Sid. This means that in such studies, the effects of the random elements of the intervention on the sham group should be considered [33].

## Conclusion

This study focused on assessing the effect of auriculotherapy on nurses’ occupational cognitive failure. The findings demonstrated that applying pressure at specific points, such as shen men, zero points, the hippocampus, the master cerebral cortex, the brain, and memory 1 and 2, over a period of 6 weeks reduced the severity of cognitive failure in nurses. Furthermore, the effects of the intervention were observed to persist for at least one month.

### Implications for practice and future directions

Based on these results, it can be concluded that ear acupressure represents an accessible, safe, noninvasive, and cost-effective treatment approach that can assist nurses in experiencing symptoms of occupational cognitive failure. However, it is suggested that future studies should be conducted using more objective assessment scales such as meridian energy to strengthen the evidence. In addition, a smaller number of ear acupuncture points should be used in future studies for stronger internal validity.

### Limitations of the study

A daily note sheet was utilized to mitigate recall bias and uncertainties regarding the nurses’ function in the intended intervention during the study; this sheet included detailed information, as described in the methods section. However, these factors are limitations of the study. The fact that the participants were nurses and potentially knew how to perform auriculotherapy may have compromised the blinding of the study. Additionally, allocating the entire sample to nurses with moderate cognitive failure could impact the generalizability of the findings. Therefore, caution should be exercised when generalizing the results to individuals with mild or severe cases of cognitive failure. This study only collected subjective data (questionnaires), and there was insufficient measurement of the empirical data. It is recommended that future studies consider the limitations of the current study. Most of the nurses studied worked in the emergency department, which may influence the results of the study.

### Electronic supplementary material

Below is the link to the electronic supplementary material.

Supplementary Material 1

## Data Availability

The corresponding author will make the data available upon responsible request.
